# Comparison of treatment outcomes of adolescents on HIV treatment before and during the coronavirus disease 2019 pandemic in Cape Town, South Africa: A retrospective cohort study

**DOI:** 10.4102/hsag.v31i0.3233

**Published:** 2026-03-13

**Authors:** Nazreen Khan, Brian van Wyk, Talitha Crowley

**Affiliations:** 1 School of Public Health, Faculty of Community and Health Sciences, University of the Western Cape, Cape Town, South Africa; 2 School of Nursing, Faculty of Community and Health Sciences, University of the Western Cape, Cape Town, South Africa

**Keywords:** adolescents, HIV, COVID-19, viral load suppression, retention in care, antiretroviral therapy, South Africa

## Abstract

**Background:**

The coronavirus disease 2019 (COVID-19) pandemic disrupted healthcare systems, posing risks for adolescents living with HIV (ALHIV) in resource-limited, high HIV-prevalence settings. These disruptions threatened antiretroviral therapy (ART) adherence, viral load suppression (VLS) and retention in care (RiC).

**Aim:**

This study aimed to compare treatment outcomes of ALHIV on ART in the Khayelitsha and Eastern Substructure (KESS) before and during the COVID-19 pandemic.

**Setting:**

The study was performed in KESS, Cape Town, South Africa.

**Methods:**

A retrospective cohort analysis was conducted among ALHIV aged 10–19 years receiving ART at public health facilities, pre-COVID-19 (before 01 March 2020) and during COVID-19 (01 March 2020–31 December 2021). Sociodemographic, clinical, and treatment data were analysed. Descriptive and inferential statistics compared outcomes and determined factors associated with VLS (< 1000 copies/mL) using SPSS v.30.

**Results:**

Data from 1702 ALHIV (pre-COVID-19) and 2733 ALHIV (during COVID-19) were analysed. Viral load suppression declined from 82.1% to 64.8%, while full VLS (< 50 copies/mL) from 70.8% to 53.7% (*p* = 0.065). Antiretroviral therapy adherence fell from 96.4% to 70.0% (*p* < 0.001), and RiC 80.3% to 76.3% (*p* < 0.001). In multivariate analysis, higher CD4 count, and consistent ART adherence predicted VLS.

**Conclusion:**

Antiretroviral therapy adherence and VLS rates among ALHIV declined during COVID-19. Adolescent-centred healthcare delivery models are needed to ensure continuity of HIV treatment during public health emergencies.

**Contribution:**

This study provides local evidence on the pandemic’s impact in a high-burden South African context. By quantifying declines in ART adherence, RiC, and VLS, it highlights ALHIV vulnerabilities and the need to strengthen adolescent-responsive, resilient healthcare systems.

## Introduction

Adolescents aged 10–19 years old living with HIV are a particularly vulnerable group because of developmental, social and healthcare-related challenges (Baltag, Guthold & Sawyer 2020; World Health Organization 2024). In 2023, over one million adolescents aged 15–19 years old were living with HIV globally, with approximately 840 000 in sub-Saharan Africa (SSA) (UNICEF 2024). South Africa bears a significant burden, with adolescent girls disproportionately affected because of biological and socio-economic vulnerabilities (Govender et al. 2022; Human Sciences Research Council 2023).

Despite expanded access to antiretroviral therapy (ART), adolescents living with HIV (ALHIV) persist in experiencing markedly inferior treatment outcomes than adults, a trend observed across multiple studies and settings (Enane, Vreeman & Foster 2018; Tarantino, Lowery & Brown 2021). Adolescents consistently demonstrate lower rates of viral load suppression (VLS) compared to adults, with some studies reporting that only 74% of adolescents achieve VLS (< 1000 copies/mL) and just 41% reach full VLS (< 50 copies/mL), falling short of the Joint United Nations Programme on HIV/AIDS (UNAIDS) 95-95-95 targets (UNAIDS 2021). Furthermore, while younger adolescents may initially show promising immunological responses after starting ART, they are more likely to experience virological failure and detectable viral loads after 12 months of treatment initiation (Ferrand et al. 2016; Han et al. 2021). Retention in care (RiC) also presents a substantial challenge, as ALHIV, particularly those aged 15–19 years old, are at higher risk of loss to follow-up (LTFU) compared to adults, further undermining treatment success (Han et al. 2021; Molopa et al. 2024). These disparities are driven by multiple factors, including suboptimal adherence linked to developmental challenges, stigma, and mental health issues, as well as gaps in adolescent-friendly service delivery (Cluver et al. 2018; Crowley, Van der Merwe & Skinner 2021; Mayman & Van Wyk 2022; Mushy et al. 2024; World Health Organization 2013).

For instance, clinics that do not provide tailored support or optimal ART regimens, such as dolutegravir (DTG)-based therapies, report higher rates of viral non-suppression among ALHIV (Mushy et al. 2024).

Programmatic interventions incorporating peer support, psychosocial care, and community-based case management have shown promise in providing both RiC and VLS among adolescents (Rakhmanina, Foster & Agwu 2024). Collectively, these findings highlight the urgent need for developmentally appropriate, comprehensive HIV care strategies that address the unique barriers faced by adolescents in order to close the treatment outcome gap with adults.

The coronavirus disease 2019 (COVID-19) pandemic, beginning in early 2020, exacerbated these challenges. Lockdowns and service disruptions threatened ART access and continuity, especially in resource-limited settings in Africa (Mayman & Van Wyk 2022; Pillay et al. 2021). Adolescents encountered new obstacles, including clinic closures, mobility restrictions, increased mental health stressors, and diminished social support, all of which could negatively impact ART adherence and RiC (Mayman & Van Wyk 2023; Pillay et al. 2021).

While numerous studies have investigated the impact of COVID-19 on general HIV service delivery, relatively few have specifically examined its effects on adolescents, and even fewer have utilised disaggregated data within local South African contexts (Cluver et al. 2020; Mayman & Van Wyk 2023). In the Western Cape, especially within the Khayelitsha and Eastern Substructure (KESS), HIV prevalence remains disproportionately high, compounded by pronounced socioeconomic vulnerabilities, including poverty, unemployment, and informal housing, that exacerbate barriers to healthcare access (Statistics South Africa 2011; Stinson et al. 2017). During the COVID-19 pandemic, adolescents in this region experienced notable disruptions in care, characterised by delayed clinic visits, diminished psychosocial support, and challenges in accessing medication (Mayman & Van Wyk 2022). Despite these obstacles, differentiated service delivery (DSD) models implemented locally, such as multi-month ART dispensing and youth-friendly clinics, contributed to sustaining some continuity of care (Hightow-Weidman et al. 2020; Maskew et al. 2022).

Nonetheless, there remains an urgent need for adolescent-centred, contextually responsive interventions that address the complex interplay between structural inequalities and treatment outcomes (Crowley et al. 2021; Hlophe, Van Wyk & Mukumbang 2023). Consequently, a thorough understanding of the pandemic’s impact on adolescent HIV care in high-burden, resource-limited settings such as KESS is critical to inform strategies for health system resilience and targeted policy development (Mayman & Van Wyk 2023; Van Wyk, Kriel & Mukumbang 2020).

This study aimed to compare treatment outcomes, including VLS, ART adherence, and RiC, among ALHIV before and during the COVID-19 pandemic. The authors hypothesised that the pandemic negatively impacted these outcomes.

## Research methods and design

### Study design and setting

A retrospective cohort study was conducted utilising de-identified clinical data sourced from the Provincial Health Data Centre (PHDC) of the Western Cape Department of Health and Wellness. The study was set in KESS, which includes Khayelitsha – one of the largest townships in South Africa with a population of approximately 500 000 people and an estimated unemployment rate of 40% (City of Cape Town Strategic Development Information and GIS Department 2013; Phelanyane et al. 2023). The population is predominantly black African and characterised by high levels of poverty, informal housing, and a substantial HIV burden, evidenced by an antenatal HIV prevalence of 31% reported in 2017. The public-sector ART programme services approximately 50 000 individuals in this area, providing free HIV care (Phelanyane et al. 2023; Stinson et al. 2017).

### Study population

The study population comprised ALHIV aged 10–19 years old who received ART at public health facilities in KESS. Two cohorts were defined based on ART engagement periods: a pre-COVID-19 cohort (prior to 01 March 2020) and a during COVID-19 cohort (from 01 March 2020 to 31 December 2021). Inclusion criteria encompassed confirmed HIV diagnosis, ART initiation at a KESS public health facility, documented ART engagement within the specified timeframes, and age between 10 and 19 years old at data extraction. Records with missing critical demographic information (i.e. age and gender) or treatment outcomes data, such as VLS status, were excluded from analysis.

### Data sources and measurement

Data were extracted from the PHDC, which integrates patient-level clinical, laboratory, and pharmacy records from public health facilities across the Western Cape (Boulle et al. 2019). Variables included demographic characteristics (age and gender), clinical data (ART initiation dates and dates of last ART medication collection), and laboratory results (viral load measurements and latest CD4 count). Viral load suppression was defined in accordance with the national guidelines as viral load below 1000 copies/mL (South African National Department of Health 2020; Western Cape Government Department of Health 2018).

### Sample size

A census approach was employed, including all eligible records within the defined periods. The pre-COVID-19 cohort comprised 1702 ALHIV, while the during COVID-19 cohort included 2733 ALHIV. No formal sample size calculation was conducted given the retrospective nature of the study and comprehensive inclusion of available data.

### Statistical analysis

Descriptive statistics were used to summarise cohort characteristics. Categorical variables were presented as frequencies and percentages, while continuous variables were reported as means with standard deviations or medians with interquartile ranges, depending on their distribution. Comparisons between pre-COVID-19 and during COVID-19 cohorts were performed using Chi-square tests for categorical variables.

The primary outcome of interest was VLS, defined as a viral load of less than 1000 copies/mL (South African National Department of Health 2020; Western Cape Government Department of Health 2018). To identify independent predictors of VLS during the COVID-19 period, multivariate logistic regression analysis was performed. Predictor variables incorporated into the model included age group (10–14 years versus 15–19 years), gender (male versus female), CD4 count category (< 200, 200–349, ≥ 350 cells/mm^3^), ART regimen (first-line versus second-line or other), duration on ART (< 12 months, 13–23 months, ≥ 24 months), ART adherence status (adherent versus non-adherent), and RiC (retained versus not retained). Selection of variables for inclusion was guided by clinical relevance and results from bivariate analyses. Adjusted odds ratio (AOR) with corresponding 95% confidence intervals (CIs) were reported for each predictor.

Statistical significance was set at a two-sided *p*-value of less than 0.05. All statistical analyses were conducted using SPSS v.30. Missing data were addressed through listwise deletion in multivariate analyses. The extent and distribution of missing data for each variable are presented in Table 1.

**TABLE 1:** Characteristics of adolescents on HIV treatment in pre-coronavirus disease 2019 (before 01 March 2020) and during coronavirus disease 2019 (01 March 2020–31 December 2021).

Characteristics		Pre-COVID-19 (< 01 March 2020) (*N* = 1702)		During COVID-19 (01 March 2020–31 December 2021) (*N* = 2733)		*p*-value
		*n*	%	*n*	%	
Age (in years)	10–14	705	41.4	889	32.5	< 0.001
	15–19	997	58.6	1844	67.5	
Sex	Female	970	57.0	1812	66.3	< 0.001
	Male	726	42.7	907	33.2	
	Unknown	6	0.4	14	0.5	
CD4 count (in cells/mm^3^)†	< 200	11	0.6	141	5.2	< 0.001
	200–349	13	0.8	235	8.6	
	≥ 350	524	30.8	851	31.1	
	Missing	1154	67.8	1506	55.1	
Duration on ART (in months)	< 12	74	4.3	570	20.9	< 0.001
	13–23	75	4.4	199	7.3	
	≥ 24	1544	90.7	1964	71.9	
	Missing	9	0.5	-	-	
ART regimen	First-line	1441	84.7	1939	70.9	< 0.001
	Second-line	200	11.8	552	20.2	
	Other	61	3.6	242	8.9	
Medication adherence	Yes	1640	96.4	1914	70.0	< 0.001
	No	62	3.6	819	30.0	
Viral load† (in copies/mL)	Fully suppressed (< 50)	1205	70.8	1466	53.7	0.065
	Suppressed (< 1000)	192	11.3	304	11.1	
	Unsuppressed (≥ 1000)	272	16.0	331	12.1	
	Missing	33	1.9	632	23.1	
Retained in care	Yes	1367	80.3	2085	76.3	< 0.001
	No	335	19.7	648	23.7	

ART, antiretroviral therapy; COVID-19, coronavirus disease 2019.

† , Only CD4 count and viral load suppression were calculated based on test results that had valid CD4 and viral load test dates. These dates were adjusted to align with their respective time periods (pre-COVID-19 and during COVID-19).

### Bias

Potential sources of bias include missing laboratory data and misclassification errors inherent to routine health records. To mitigate these, comprehensive data cleaning and validation procedures were implemented. Nonetheless, residual confounding because of unmeasured variables cannot be excluded.

### Ethics considerations

An application for full ethical approval was made to the University of the Western Cape Biomedical Research Ethics Committee and ethics consent was received on 18 July 2025. The ethics approval number is BM25/1/13. To maintain the confidentiality of human participants, the study utilised de-identified secondary data sourced from the PHDC, as authorised by their data governance policies. All data were securely stored on password-protected servers, with access strictly limited to authorised members of the research team. These measures, in adherence to institutional and provincial data governance frameworks, ensured the protection of patient confidentiality and the integrity of the data throughout the study.

## Results

Table 1 presents the comparison of sociodemographic, clinical and treatment characteristics of ALHIV on ART in the period pre-COVID-19 and during the COVID-19 pandemic. The cohort assessed during the COVID-19 pandemic exhibited a moderately higher proportion of females (57.0% before COVID-19 versus 66.3% during COVID-19) and a lower representation of adolescents aged 10–14 years old (32.5% versus 41.4%) relative to the pre-pandemic cohort. These demographic differences are detailed in Table 1. A marginal decrease was observed in the proportion of ALHIV with CD4 counts ≥ 350 cells/mm^3^ (30.8% during COVID-19 versus 31.1% pre-COVID-19). Notably, adherence to ART demonstrated a substantial decline from 96.4% before the pandemic to 70.0% during the COVID-19 period (*p* < 0.001). RiC similarly decreased from 80.3% to 76.3% (*p* < 0.001).

The recorded prevalence rates for VLS (defined as viral load less than 1000 copies/mL) – were reduced from 82.1% pre-COVID-19 to 64.8% during COVID-19; although not statistically significant (*p* = 0.065).

**FIGURE 1:**
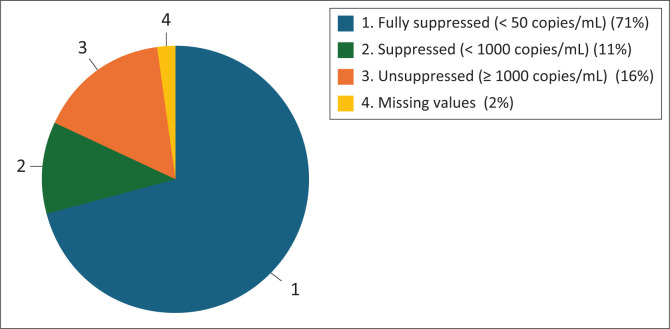
Viral load suppression rates of adolescents living with HIV on antiretroviral therapy in the Khayelitsha and Eastern Substructure before the coronavirus disease 2019 pandemic (*N* = 1702).

**FIGURE 2:**
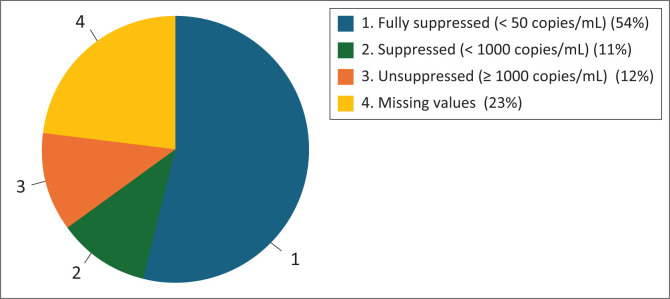
Viral load suppression rates of adolescents living with HIV on antiretroviral therapy in the Khayelitsha and Eastern Substructure during the coronavirus disease 2019 pandemic (*N* = 2733).

Furthermore, full VLS (defined as a viral load below 50 copies/mL) decreased from 70.8% to 53.7% (Table 1). Figure 1 and Figure 2 visually depict these trends, illustrating the decline in VLS rates before and during the pandemic, while also pointing out that a notable proportion of adolescents continued to achieve full VLS despite the challenges posed by the COVID-19 pandemic.

Multivariate regression analysis was conducted on identified factors independently associated with VLS during the COVID-19 period. The results of both crude odds ratio (COR) and AOR are summarised in Table 2. Higher CD4 counts were strongly predictive of VLS. This is demonstrated by the finding that adolescents with higher CD4 counts had significantly lower odds of viral non-suppression, with those in the ≥ 350 cells/mm^3^ category having a notably reduced odds (AOR = 0.140; 95% CI: 0.092–0.212) compared to adolescents with CD4 counts < 200 cells/mm^3^. Antiretroviral therapy regimen was also a significant predictor: individuals on second-line (AOR = 3.237; 95% CI: 2.346–4.467) and other regimens (AOR = 3.513; 95% CI: 2.372–5.202) were more likely to achieve VLS relative to those on first-line ART.

**TABLE 2:** Determinants of viral load suppression during the coronavirus disease 2019 period in adolescents living with HIV on antiretroviral therapy in Khayelitsha and Eastern Substructure.

Characteristics	Total (Crude OR) (*N* = 2091)	Crude OR	95% CI	Total (AOR) (*N* = 2009)	Adjusted OR	95% CI
**Age (in years)**						
10–14	772	Ref	-	754	Ref	-
15–19	1319	1.101	0.861–1.408	1255	1.002	0.750–1.338
**Gender**						
Male	740	Ref	-	720	Ref	-
Female	1351	0.956	0.749–1.221	1289	1.103	0.837–1.454
**CD4 count (in cells/mm^3^)**						
< 200	124	Ref	-	124	Ref	-
200–349	231	0.413	0.263–0.650	231	0.464	0.286–0.753
≥ 350	1654	0.139	0.095–0.203	1654	0.140	0.092–0.212
**Duration on ART (in months)**						
< 12	214	Ref	-	191	Ref	-
12–23	151	0.395	0.218–0.715	137	0.514	0.267–0.988
≥ 24	1726	0.558	0.397–0.782	1681	0.642	0.418–0.986
**ART regimen**						
First-line	1454	Ref	-	1383	Ref	-
Second-line	440	2.510	1.913–3.293	436	3.237	2.346–4.467
Other	197	3.340	2.365–4.717	190	3.513	2.372–5.202
**Medication adherence**						
No	370	Ref	-	350	Ref	-
Yes	1721	0.336	0.258–0.437	1659	0.321	0.204–0.505
**Retention in care**						
No	246	Ref	-	231	Ref	-
Yes	1845	0.334	0.248–0.451	1778	1.148	0.680–1.938

Note: The total *N* is lower than in previous tables due to an additional data cleaning step that excluded viral load and CD4 count results that fell outside the during-COVID-19 period (01 March 2020–31 December 2021). Test results without valid viral load or CD4 test dates were also excluded, contributing to the reduced sample size.

COVID-19, coronavirus disease 2019; OR, odds ratio; AOR, adjusted odds ratio; CI, confidence interval; ART, antiretroviral therapy; Ref, reference category.

Optimal ART adherence emerged as a key determinant of VLS, with adherent adolescents demonstrating significantly higher odds of VLS (AOR = 0.321; 95% CI: 0.204–0.505). Although RiC was significantly associated with VLS in unadjusted analyses, this association was not statistically significant in the multivariate model (AOR = 1.148; 95% CI: 0.680–1.938).

It is important to note that the sample size for the regression analyses was reduced because of the exclusion of viral load and CD4 count results that either fell outside the defined COVID-19 period (01 March 2020–31 December 2021) or lacked valid test dates. This data cleaning step is detailed in the note beneath Table 2.

## Discussion

This study compared treatment outcomes of ALHIV receiving ART before and during the COVID-19 pandemic in KESS of Cape Town, South Africa. By analysing data from both periods, the authors aimed to assess the impact of the pandemic on key indicators, including ART adherence, RiC, and VLS. Our objective was to elucidate the extent to which pandemic-related disruptions influenced adolescent HIV care within this high-burden urban context.

The findings reveal a marked deterioration in measured treatment outcomes during the COVID-19 pandemic period, with ART adherence experiencing the most substantial decline. Specifically, ART adherence, as measured through pharmacy refill records, decreased from 96.4% pre-pandemic to 70.0% during the pandemic (*p* < 0.001). This significant reduction is consistent with existing literature that highlights the compounded challenges introduced by the pandemic, such as healthcare facility closures, restricted mobility because of lockdown regulations, limited transportation, and increased psychosocial stressors (Mayman & Van Wyk 2023; Pillay et al. 2021). Adolescents, already at heightened risk because of their developmental stage, may have faced particular difficulty maintaining consistent ART adherence under these adverse circumstances.

Retention in care also declined, albeit to a lesser extent, from 80.3% to 76.3% (*p* < 0.001). Although this reduction was less pronounced than that observed for ART adherence, even modest disruptions to care continuity are concerning for a population requiring lifelong ART. The observed decline likely reflects known barriers to sustained engagement in HIV care during the pandemic, including fears of COVID-19 infection, reduced clinic hours, and staff shortages (Jardim, Zamani & Akrami 2022; Mathamo et al. 2022).

Viral load suppression, a critical indicator of treatment efficacy, also worsened during the pandemic. Full VLS (< 50 copies/mL) decreased from 70.8% to 53.7%, and overall VLS (< 1000 copies/mL) fell from 82.1% to 64.8%. While the decline in full VLS did not reach statistical significance (*p* = 0.065), the 17.1% percentage-point reduction is clinically meaningful and indicates that fewer adolescents achieved optimal virological control during the pandemic period. Notably, these findings contrast with reports from other SSA settings where VLS remained stable, potentially because of more robust implementation of DSD models (Carpenter et al. 2024; Mugo et al. 2024). The findings from our study are displayed in Table 1; Figure 1 and Figure 2.

Multivariate regression analysis identified several factors associated with improved virological outcomes, which are likely protective factors against viral non-suppression. Higher latest CD4 counts were strongly predictive of VLS, as adolescents with CD4 counts ≥ 350 cells/mm^3^ had significantly lower odds of viral non-suppression (AOR = 0.140; 95% CI: 0.092–0.212) compared to those with CD4 counts < 200 cells/mm^3^. This aligns with findings from other studies in SSA, where higher immunological status has consistently been associated with better VLS (Ferrand et al. 2016; Han et al. 2021). Similarly, optimal ART adherence was a key determinant, consistent with previous evidence demonstrating that ART adherence is critical for achieving VLS among ALHIV (Cluver et al. 2020; Mayman & Van Wyk 2023). Conversely, being on a second-line or other ART regimen was associated with increased odds of viral non-suppression, reflecting the challenges often seen with treatment-experienced ALHIV, including prior treatment failure and more complex ART regimens (Mugo et al. 2024). While RiC was significant in bivariate analysis, it was not retained in the multivariate model, suggesting that its effect may be mediated through ART adherence or other clinical factors, a pattern observed in prior studies from South Africa, which has demonstrated protective effects of good, consistent adherence and the use of well-tolerated regimens, such as those based on dolutegravir (DTG), on virological outcomes (Elashi & Van Wyk 2022; Okonji et al. 2021; Western Cape Government Department of Health 2018).

Despite the introduction of DSD strategies during the pandemic, including multi-month dispensing and community-based ART delivery, our findings suggest that these adaptations did not fully mitigate the negative impact of the pandemic on adolescent HIV care. Vulnerable subgroups, such as younger adolescents 10–14 years old, males, and those from socioeconomically disadvantaged backgrounds, may not have benefitted equally from these interventions. This underscores the need for adolescent-responsive care models that are sensitive to age, gender, and social context (Mathamo et al. 2022; Van Wyk & Davids 2019).

Several limitations should be acknowledged. Firstly, the reliance on routinely collected electronic health data introduces the potential for measurement bias because of missing or incomplete records. Notably, CD4 count, and viral load data were more frequently incomplete during the pandemic, likely reflecting reduced laboratory capacity and fewer clinic visits, which may have reduced the power to detect significant differences. Secondly, the use of pharmacy refill data as proxy for adherence to ART may overestimate actual medication intake, as it does not confirm actual medication ingestion. Thirdly, the retrospective observational design of this study limits causal inference; while temporal associations with the pandemic are evident, unmeasured confounders such as household stressors, mental health, and social support may have influenced outcomes. Lastly, the study’s focus on a single urban district may limit the generalisability of these findings to other regions or rural settings with different healthcare infrastructures.

This analysis provides important insights into the ways in which a global public health emergency can disrupt adolescent HIV treatment outcomes in a high-burden urban setting. The observed declines in ART adherence, RiC, and VLS point out the need for adolescent-responsive, resilient care models that can sustain engagement and virological control during future health system shocks. Strengthening these systems is essential to safeguard the health and well-being of ALHIV in the face of ongoing and emerging challenges.

### Recommendations

It is recommended that DSD be strengthened by expanding multi-month ART dispensing and community-based models to improve access and adherence during disruptions. Psychosocial support should be enhanced to address mental health and social challenges exacerbated by public health emergencies. Data systems must be improved to ensure robust monitoring and to identify and address gaps in adolescent HIV care. Finally, gender-sensitive interventions should be prioritised, especially targeting adolescent girls who remain disproportionately affected.

## Conclusion

During the COVID-19 pandemic, declines were observed in ART adherence, RiC, and VLS among ALHIV. Although a substantial portion of adolescents maintained full VLS, the overall reductions underscore the heightened vulnerability of this population’s treatment outcomes during periods of systemic disruption.

Factors positively associated with achieving VLS included initiation and maintenance on a first-line ART regimen, consistent ART adherence, and higher CD4 cell counts. These findings emphasise the imperative for adaptive, adolescent-responsive models of HIV care that are resilient to public health emergencies. To safeguard treatment outcomes for adolescents in future crises, it will be essential to strengthen DSD approaches, enhance psychosocial support mechanisms, and address persistent structural barriers to care.
